# Sleep Behaviors and the Shape of Subcortical Brain Structures in Children with Overweight/Obesity: A Cross-Sectional Study

**DOI:** 10.1007/s12098-024-05094-1

**Published:** 2024-04-04

**Authors:** Cristina Cadenas-Sanchez, Jairo H. Migueles, Lucia V. Torres-Lopez, Juan Verdejo-Román, David Jiménez-Pavón, Charles H. Hillman, Andrés Catena, Francisco B. Ortega

**Affiliations:** 1 Department of Physical Education and Sports, Faculty of Sport Sciences, Sport and Health University Research Institute (iMUDS), University of Granada, Granada, Spain; 2 CIBER de Fisiopatología de la Obesidad y Nutrición (CIBEROBN), Instituto de Salud Carlos III, Granada, Spain; 3 Department of Personality, Assessment & Psychological Treatment, Mind, Brain and Behavior Research Center (CIMCYC), University of Granada, Granada, Spain; 4 MOVE-IT Research Group, Department of Physical Education, Faculty of Education Sciences University of Cádiz, Cádiz, Spain; 5 Biomedical Research and Innovation Institute of Cádiz (INiBICA) Research Unit, Puerta del Mar University Hospital University of Cádiz, Cádiz, Spain; 6 CIBER of Frailty and Healthy Aging (CIBERFES) Madrid, Madrid, Spain; 7 Department of Psychology, Northeastern University, Boston, USA; 8 Department of Physical Therapy, Movement, & Rehabilitation Sciences, Northeastern University, Boston, USA; 9 School of Psychology, University of Granada, Granada, Spain; 10 Faculty of Sport and Health Sciences, University of Jyväskylä, Jyväskylä, Finland

**Keywords:** Brain shapes, Grey matter, Obesity, Sleep, Actigraphy

## Abstract

**Objectives:**

To examine the relationship between sleep and subcortical brain structures using a shape analysis approach.

**Methods:**

A total of 98 children with overweight/obesity (10.0 ± 1.1 y, 59 boys) were included in the cross-sectional analyses. Sleep behaviors (i.e., wake time, sleep onset time, total time in bed, total sleep time, sleep efficiency, and wakening after sleep onset) were estimated with wrist-worn accelerometers. The shape of the subcortical brain structures was acquired by magnetic resonance imaging. A partial correlation permutation approach was used to examine the relationship between sleep behaviors and brain shapes.

**Results:**

Among all the sleep variables studied, only total time in bed was significantly related to pallidum and putamen structure, such that those children who spent more time in bed had greater expansions in the right and left pallidum (211–751 voxels, all *p*’s <0.04) and right putamen (1783 voxels,* p* = 0.03).

**Conclusions:**

These findings suggest that more time in bed was related to expansions on two subcortical brain regions in children with overweight/obesity.

**Supplementary Information:**

The online version contains supplementary material available at 10.1007/s12098-024-05094-1.

## Introduction

Having healthy sleep hygiene during childhood, which is characterized by attaining adequate sleep duration (i.e., 9–11 h per night), good sleep quality, maintaining appropriate sleep timing, and the absence of sleep disorders, is vital for healthy growth and development [1]. Yet, in the last decade, almost one-third of the pediatric population report sleeping eight or fewer hours per night, and having inadequate sleep behaviors (i.e., short sleep duration, poor sleep quality, long sleep latency, and irregular total time in bed) [2]. This inadequate sleep hygiene has been associated with many adverse brain health outcomes [3–5]. Indeed, such associations might be especially relevant for children with overweight/obesity, since they often have poorer sleep behaviors than normal weight children [6, 7].

A mounting body of evidence in recent years has shown a link between sleep behaviors and brain health [1, 3, 8]. Specifically, a systematic review highlighted the importance of sleep behaviors for brain development in children and adolescents [1]. However, no firm conclusions were drawn because of the heterogeneity of sleep variables and outcomes reported [1]. More recently, in this cohort, the authors have demonstrated that waking up earlier, and experiencing less time and number of occurrences of wakening after sleep onset (WASO) were associated with higher gray matter volume, using a whole brain volumetric approach [3]. Riggins and colleagues observed that habitual total sleep duration predicted hippocampal head subfield volumes in younger children [8].

Previous studies have mainly focused on brain volumetric analyses as indicators of brain health [9, 10]. However, the shape of the subcortical structures has been recently proposed as another brain health indicator worth of investigation [11]. Indeed, the changes in the morphology of the subcortical brain structures (i.e., expansions or contractions in specific regions of the subcortical structures) is a more sensitive method than overall brain volume analysis which can be precisely detected using magnetic resonance imaging (MRI). Shape analyses may shed light on the relationship of sleep behaviors and brain health by providing more accurate information on brain morphology. Therefore, the aim of this study was to examine the association between sleep behaviors and subcortical brain structures using a shape analysis approach. The authors hypothesized that children with good sleep hygiene practices are more likely to present greater expansions in brain shapes.

## Material and Methods

This study is under the framework of ActiveBrains project (http://profith.ugr.es/activebrains), a randomized controlled trial, which aimed of examining the effects of an exercise intervention on brain health in children with overweight/obesity (NCT02295072) [12]. Briefly, the inclusion criteria were: children aged 8–11 y, right handed, presenting overweight or obesity based on the World Obesity Federation’s cut-offs [13, 14], without psychological or physical disorders, and for girls, not having onset of menarche. Therefore, from the 110 children who enrolled in the project, those with valid data at baseline were included in this cross-sectional study (N = 98, 10.0 ± 1.1 y, 59 boys). This study was powered for the main article of the project aiming to detect small- to medium-effects in brain health outcomes, with an alpha error of 5% and a power of 80%, in an exercise-based randomized controlled trial [12, 15], and therefore was not specifically powered for this cross-sectional secondary analyses. Parents or legal guardians were informed of the study and written informed consent was obtained. The ActiveBrains project was approved by the Human Research Ethics Committee of the University of Granada.

Participants were asked to wear accelerometers (ActiGraph, Pensacola, FL, USA) on their non-dominant wrist for 7 consecutive days (24 h/d). They also were required to complete a sleep log with information about wake and sleep onset times each day. Detailed information on accelerometer data processing can be found elsewhere [3]. The algorithm developed by Sadeh et al. was applied [16].

Sleep behaviors included sleep timing (i.e., wake time and sleep onset), sleep duration (i.e., total time in bed and total sleep time) and sleep patterns (i.e., sleep efficiency, WASO time and number of WASO). Total time in bed was derived as the time difference between wake time and sleep onset time. Total sleep time represents the sum of all minutes classified as sleep within total time in bed. Sleep efficiency is the percentage of time classified as sleep over the total time in bed. The authors also derived the total amount of time classified as WASO time and the number of WASO every night.

High resolution T1-weighted images were collected by MRI (Siemens Magnetom Tim Trio, 3 T, Siemens, Germany) [17]. The duration of the gradient-echo sequence of the T1 was 6 min and 34 s for each child. FIRST (http://fsl.fmrib.ox.ac.uk/fsl/fslwiki/FIRST) command was used by the FSL software for segmentation of subcortical brain structures and shape analysis. FIRST is a model-based registration tool that uses a set of fifteen subcortical structures: brain stem, and right and left hemispheres of pallidum, putamen, thalamus, nucleus accumbens, amygdala, caudate nucleus, and hippocampus.

Shape analysis is focused on the individual mesh (aligned to Montreal Neurological Institute) composed of a large number of vertices and triangles. The number of vertices and triangles is the same for each structure, which allows for comparison within and between each participants’ vertex. The rotation and translation are removed. Vertex-wise analysis methods have been previously described [18, 19]. The authors used the radial distance of each vertex to the medial curve of the structure (the centroid curve of the structure boundary in each section) for assessing local changes in each structure. Regional expansions and contractions (i.e., radial distances related each vertex spatial location to the core line of the structure) of the structure were the indicators of local changes in the structure shape [11].

Peak height velocity was derived from standing and sitting height as a continuous measure of maturational status [20]. Parental educational level was self-reported.

Characteristics of the study participants (mean and standard deviation or percentages) were reported using descriptive and frequency statistics. In all analyses, boys and girls were analyzed together, as no significant sex interactions were observed. Additionally, the authors performed an analysis of interaction by body mass index, but no significant interaction was found, and thus, it has not been included in the analysis. In order to test the correlation between sleep outcomes, bivariate Pearson correlation tests were performed among the sleep-related behaviors. To examine the relationship between sleep behaviors and subcortical brain structures, partial correlation permutation approach was applied. For these analyses, in order to test the independency between the sleep behaviors studied, all sleep variables were introduced together with the confounders (i.e., sex, peak height velocity, parental education) as covariates. Sleep efficiency and number of WASO were excluded from the final model due to different reasons: i) sleep efficiency is an outcome derived from total time in bed and total sleep time, ii) number of WASO is a variable strongly correlated to WASO time, and iii) sleep efficiency and number of WASO were strongly correlated to each other (r >0.9), which increased the risk for multicollinearity in the model. As exploratory analyses, the authors further tested the relationship of sleep behaviors and brain shapes using a different partial correlation permutation model for each sleep behavior excluding the rest of sleep behaviors from the model.

In order to account for multiple testing, the authors used the threshold-free cluster enhancement approach (TFCE) [21]. Results are displayed as color-coded significance maps. They used blue color to indicate a negative outcome-predictor association (i.e., higher the value of the predictor, the smaller the radial distances), orange color indicated positive relationships (higher the value of the predictor, the larger the radial distances) and gray color to indicate non-significant associations. For all analyses, the *P* value was set at α <0.0 TFCE corrected.

## Results

Table 1 shows the descriptive characteristics of the study sample. Descriptive characteristics for subcortical brain structure volumes for the total and the left and right hemispheres can be found in Table 2. Supplementary Table S1 shows bivariate correlation among the sleep variables.

**Table 1:** Descriptive characteristics of the study sample

	All (n = 98)	Boys (n = 59)	Girls (n = 39)
Age (years)	10.0 ± 1.1	10.2 ± 1.1	9.9 ± 1.1
Weight (kg)	55.8 ± 10.8	56.3 ± 10.5	54.9 ± 11.2
Height (cm)	144.0 ± 8.2	144.6 ± 7.4	143.2 ± 9.2
Body mass index	26.7 ± 3.6	26.8 ± 3.7	26.6 ± 3.4
Weight status (n, %)*			
Overweight	25, 25.5	16, 27.1	9, 23.1
Obesity type 1	43, 43.9	27, 45.8	16, 41.0
Obesity type 2	20, 20.4	10, 16.9	10, 25.6
Obesity type 3	10, 10.2	6, 10.2	4, 10.3
Peak height velocity (years)	12.3 ± 0.7	12.8 ± 0.4	11.6 ± 0.3
Parental university level (n, %)			
Neither parent	65, 66.3	42, 71.2	23, 59.0
One parent	17, 17.3	10, 16.9	7, 17.9
Both parents	16, 16.3	7, 11.9	9, 23.1
**Sleep behaviors**^**a**^			
Wake time (hh:mm)	8:07 ± 0:34	8:05 ± 0:33	8:10 ± 0:35
Sleep onset time (hh:mm)	23:02 ± 0:39	23:01 ± 0:40	23:04 ± 0:38
Total time in bed (min/d)	527.5 ± 31.6	526.6 ± 33.4	528.9 ± 29.1
Total sleep time (min/d)	458.5 ± 34.9	455.8 ± 32.3	462.4 ± 38.6
Sleep efficiency (%)	84.6 ± 4.9	84.0 ± 4.4	85.5 ± 5.6
WASO time (min/d)	76.7 ± 24.0	79.8 ± 20.3	71.8 ± 28.3
WASO number (nr.)	23.3 ± 4.5	24.1 ± 4.1	22.2 ± 5.0

Data are presented as mean ± standard deviation unless otherwise stated

*WASO* Wakening after sleep onset

^*^Classified according to Cole and Lobstein [13] and Bervoets and Massa [14]

^a^Classified according to Sadeh et al. [16] cut-off points for non-dominant wrist

**Table 2:** Descriptive characteristics of the subcortical brain structures (volume, mm^3^)

	**Total**	**Left** **hemisphere**	**Right** **hemisphere**
Brain stem	19649 ± 1920	-	-
Pallidum	3299 ± 312	1652 ± 160	1647 ± 162
Putamen	9929 ± 1029	4944 ± 498	4984 ± 561
Thalamus	15614 ± 1298	7864 ± 643	7750 ± 672
Nucleus accumbens	1005 ± 191	542 ± 113	462 ± 102
Amygdala	2651 ± 375	1317 ± 184	1332 ± 239
Caudate nucleus	7629 ± 866	3796 ± 434	3833 ± 510
Hippocampus	7048 ± 680	3462 ± 374	3585 ± 385

Data are presented as mean ± standard deviation

**Fig. 1:**
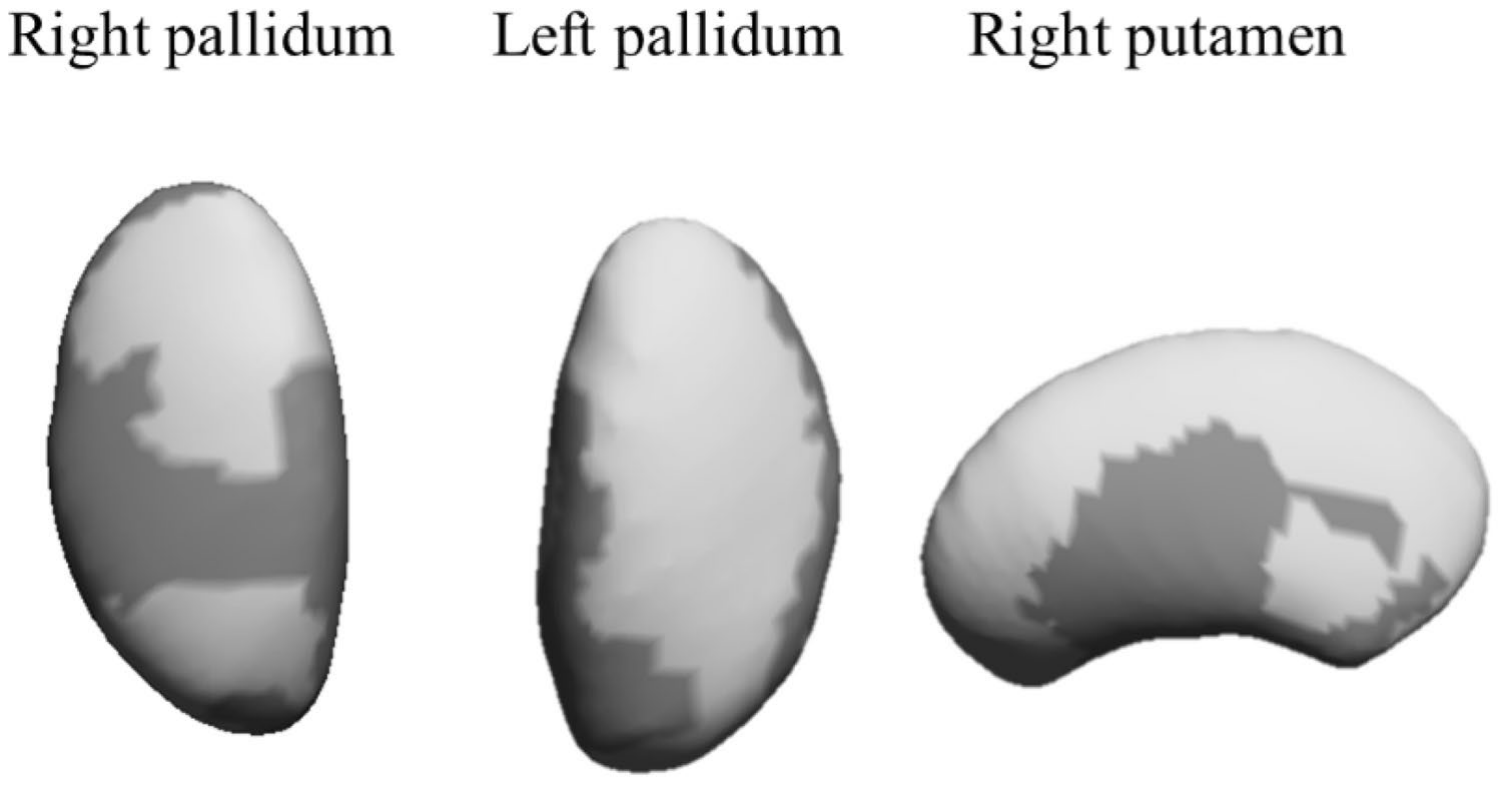
Mappings of significant expansions of subcortical brain structures related to total time in bed. The color indicates the significance threshold-free cluster enhancement corrected *P* values, with light gray depicting significant positive association between sleep variables and brain structures. Dark gray indicates no association. All the analyses were adjusted for sex, peak height velocity, parental education, and all sleep variables used for the analyses (i.e., wake time, sleep onset time, total sleep time, and wakening after sleep onset time)

Vertex-wise permutation tests for sleep behaviors and subcortical brain structures adjusted for basic confounders (i.e., sex, peak height velocity, parental education, and wake time, sleep onset time, total time in bed, total sleep time, and WASO time) are shown in Table 3. Out of all the sleep variables studied, total time in bed was the only significant variable positively associated (i.e., greater expansions) with two subcortical brain structures: right and left pallidum (211 to 751 voxels, all *p*’s <0.04), and right putamen (1783 voxels, *p* = 0.03). No significant correlations were observed for the remaining subcortical brain structures analyzed (i.e., brain stem, thalamus, nucleus accumbens, amygdala, caudate nucleus, and hippocampus) (all *p*’s >0.05). Figure 1 depicts these associations by means of a color-coded significance map.

**Table 3:** Brain regions showing a significant correlation between sleep behaviors and subcortical brain structures, while controlling for all other sleep variables

	**Contrast**	**Voxels**	***p***
***Total time in bed***			
Right pallidum	Expansions	346	0.037
		211	0.034
Left pallidum	Expansions	751	0.038
Right putamen	Expansions	1783	0.031

Positive associations (i.e., expansions) indicate larger radial distance in the structures studied (*p* <0.05, threshold-free cluster enhancement corrected). Data were adjusted for sex, peak height velocity, parental education, and all sleep variables used for the analyses (i.e., wake time, sleep onset time, total sleep time, and wakening after sleep onset time)

In exploratory analyses, the authors further examined the correlation between sleep variables and subcortical brain structures in different models, adjusted only for sex, peak height velocity, and parental education (Supplementary Table S2). Wake time positively related (i.e., greater expansions) to right and left amygdala (166 to 662 voxels, all *p*’s ≤0.019). Sleep onset time was negatively associated (i.e., contractions) with right and left putamen (480 to 1724 voxels, all *p*’s ≤0.038), and positively related to right and left amygdala (175 to 683 voxels, all *p*’s ≤0.027). Total time in bed was positively related to several structures (right and left pallidum, right and left putamen, and right thalamus; 932 to 2882 voxels, all *p*’s ≤0.018) and negatively related to left caudate nucleus (801 voxels, *p* = 0.002). WASO time was negatively related to right nucleus accumbens (13 voxels, *p* = 0.039) but positively related to right caudate nucleus (69 to 250 voxels, all *p*’s ≤0.046). No significant associations were found with the remaining sleep variables studied (all *p*’s >0.05).

## Discussion

The present findings indicate that more time in bed is related to specific expansions in the right and left pallidum, and the right putamen in children with overweight/obesity. However, no significant correlations were observed between sleep variables and the other subcortical brain structures, i.e., brain stem, thalamus, nucleus accumbens, amygdala, caudate nucleus, and hippocampus.

The present study contributes to the existing literature by studying the relationship between sleep behaviors and the morphology of subcortical brain structures in children. Specifically, the authors observed that for children with overweight/obesity, higher total sleep duration was related to greater expansions in the pallidum and putamen. No previous studies examining these associations in healthy populations or children were found, which hampers the comparisons of present findings with other studies. Replication of these findings in future studies is warranted. To the best of authors’ knowledge, only two studies in adults investigated the association between the shape of subcortical brain structures and sleep in insomnia patients [22, 23]. The first study tested the potential structural alterations of the amygdala in adults with insomnia compared with healthy controls with adequate sleep [22]. The second study examined the associations of the shape of subcortical brain structures with sleep in adults with insomnia [23]. The conclusions from these two studies suggest a potential sleep-related atrophy in the amygdala, putamen, and hippocampus. The authors cannot directly compare their findings with these studies due to different methodological reasons (actigraphy vs. self-reported questionnaire) and characteristics of the study participants (children with overweight/obesity vs. adults with insomnia). Notwithstanding, the results of the previous studies partially agree with present findings, showing a relationship between sleep behaviors and the putamen, yet the present study did not find a sleep-related correlation with amygdala and hippocampus as concluded in the previous studies [22, 23]. Likewise, they did not observe any change in thalamus and caudate nucleus in line with the observations in the present sample [23]. Although the present findings are promising with the potential to decipher the relation between sleep behaviors and brain morphology in children, caution should be paid when interpreting these findings since causal relationships cannot be stated.

When interpreting present findings, it is crucial to understand the role of the basal ganglia (i.e., caudate nucleus, pallidum, putamen, and nucleus accumbens) in sleep regulation and wakefulness. The basal ganglia is a key area involved in motor function, habit formation, reward/addictive behaviors, and executive function [24], all of which depend on wakefulness [25]. Indeed, there are reciprocal connections between the basal ganglia and every part of the sleep–wake circuit: cerebral cortex, brain stem, basal forebrain, thalamus and hypothalamus [25]. Moreover, the role of the pallidum during the sleep cycle may be reflected in the activity of the striatum, which collectively refers to the caudate nucleus, putamen and nucleus accumbens. Data from studies in mice have demonstrated changes in sleep pattern with lesioning or stimulation of the pallidum [26]. Specifically, a lesion in the pallidum decreased sleep behaviors, increased fragmentation and shortened sleep duration. In contrast, stimulation of this region was related to increases in both non-rapid eye movement and rapid eye movement sleep time [26]. Therefore, based on the evidence and confirming the present findings, it seems that the basal ganglia is a key group of structures with an important role in sleep behaviors.

To date, few previous studies suggest a beneficial link between sleep behaviors and gray matter volume in several structures in children and adolescents [3, 27, 28]. For instance, Taki et al. observed that sleep duration during weekdays affected hippocampal gray matter volume in healthy children [27]. Further, Urrila et al. concluded that shorter time in bed during weekdays correlates with smaller brain gray matter volumes in frontal, anterior cingulate, and precuneus cortex regions in adolescents [28]. The present study investigates the week as a whole using weighted averages to account for the correspondent weight of weekdays and weekend days, and thus, providing a clear perspective on the relationship between sleep behaviors and brain shapes rather than assuming different associations dependent upon the day in which sleep occurs [3]. Analyzing sleep behaviors with this approach, a recent study from the same cohort explored the associations of sleep behaviors with gray matter volume in the whole brain, with a particular focus on the hippocampus in children with overweight/obesity [3]. The main finding was that sleep behaviors were associated with gray matter volume in multiple cortical and subcortical brain structures including the hippocampus [3]. The lack of association found in the putamen and pallidum using the whole-brain approach could suggest that an unmasked potential association was detected by the brain shapes analyses, which allows for the detection of locally precise changes in brain morphology of various brain structures.

The study of the sleep-brain connection in this period of the lifespan, when the brain undergoes major developmental changes, is of public health significance. The present study provides important insight into understanding how sleep hygiene is related to brain shapes in children with overweight/obesity. While the current study did not specifically examine the impact of brain volumes on daytime behavior and intelligence, it is noteworthy that a previous study within the same cohort found a positive correlation between expansions in the right pallidum and intelligence [17]. Nevertheless, more research is needed to gain a further understanding of how the various expansions and contractions of certain subcortical structures relate to brain health. Future studies with larger and more diverse samples should aim to replicate these findings, paying particular attention to potential sex and weight status differences, as well as, exploring the broader link between brain shapes and cognition.

The limitations of this study were: i) its cross-sectional design does not allow a causal interpretation of the findings; ii) the inclusion of children with overweight/obesity limits the generalizability of these results, and iii) the use of actigraphy instead of the gold-standard, polysomnography. Moreover, the accelerometer-based estimates of sleep behaviors are an estimation based on movement patterns, and not purely sleep behaviors. As such, these findings should be taken with caution. Still, accelerometers are a non-invasive, objective, and valid technique for assessing sleep behaviors in free-living individuals [16, 29], and they also demonstrate high agreement with polysomnography [30].

Overall, these results indicate that the total time spent in bed is positively associated with further developments in the right and the left pallidum and the right putamen, suggesting a beneficial role of sleep hygiene on brain.

## Supplementary Information

Below is the link to the electronic supplementary material.

Supplementary file1 (DOCX 19 KB)

## Declarations

### Clinical Trial Registration

NCT02295072.
