# Supplementary material for: Sleep Behaviors and the Shape of Subcortical Brain Structures in Children with Overweight/Obesity: A Cross-Sectional Study
Source: Indian J Pediatr. 2024 Apr 4;92(7):703–9. doi: 10.1007/s12098-024-05094-1 (PMC12182451; doi:10.1007/s12098-024-05094-1)
Supplement: Supplementary file 1 — Supplementary file1 (DOCX 19 KB) [file 12098_2024_5094_MOESM1_ESM.docx]

**Supplementary Table S1** Bivariate correlation among the sleep behaviors

|  | **Sleep onset time** | **Total time in bed** | **Total sleep time** | **Sleep efficiency** | **WASO time** | **WASO number** |
| --- | --- | --- | --- | --- | --- | --- |
| Wake time | 0.699** | -0.486** | -0.170 | 0.202* | -0.212* | -0.221* |
| Sleep onset time |  | 0.121 | -0.281** | 0.186 | -0.099 | -0.099 |
| Total time in bed |  |  | 0.556** | 0.011 | 0.149 | 0.155 |
| Total sleep time |  |  |  | 0.756** | -0.318** | -0.582** |
| Sleep efficiency |  |  |  |  | -0.619** | -0.917** |
| WASO time |  |  |  |  |  | 0.705** |

*WASO* Wakening after sleep onset

**p* <0.05

***p* <0.01

**Supplementary Table S2** Brain regions showing a significant correlation between sleep behaviors and subcortical brain structures

|  | **Contrast** | **Voxels** | ***p*** |
| --- | --- | --- | --- |
| ***Wake time*** |  |  |  |
| Right amygdala | Expansions | 311 | 0.010 |
|  |  | 166 | 0.019 |
| Left amygdala | Expansions | 662 | 0.002 |
| ***Sleep onset time*** |  |  |  |
| Right putamen | Contractions | 1724 | 0.028 |
| Left putamen | Contractions | 633 | 0.036 |
|  |  | 480 | 0.038 |
| Right amygdala | Expansions | 293 | 0.016 |
|  |  | 175 | 0.027 |
| Left amygdala | Expansions | 683 | 0.007 |
| ***Total time in bed*** |  |  |  |
| Right pallidum | Expansions | 932 | 0.009 |
| Left pallidum | Expansions | 993 | 0.018 |
| Right putamen | Expansions | 2458 | 0.008 |
| Left putamen | Expansions | 1759 | 0.012 |
| Right thalamus | Expansions | 2882 | 0.012 |
| Left caudate nucleus | Contractions | 801 | 0.002 |
| ***WASO time*** |  |  |  |
| Right nucleus accumbens | Contractions | 13 | 0.039 |
| Right caudate nucleus | Expansions | 250 | 0.033 |
|  |  | 162 | 0.046 |
|  |  | 69 | 0.046 |

*WASO* Wakening after sleep onset

Positive associations (i.e., expansions) indicate larger radial distance, and negative associations (i.e., contractions) show shorter radial distance in the structures studied (*p* <0.05, threshold-free cluster enhancement corrected). Data were adjusted for sex, peak height velocity, and parental education.
